# Implementing a Photodocumentation Program

**DOI:** 10.1007/s10278-024-01236-1

**Published:** 2024-08-22

**Authors:** Eric K. Lai, Evan Slavik, Bessie Ganim, Laurie A. Perry, Caitlin Treuting, Troy Dee, Melissa Osborne, Cieara Presley, Alexander J. Towbin

**Affiliations:** 1 Department of Radiology, Tufts University School of Medicine, Boston, USA; 2 Department of Radiology, Cincinnati Children’s Hospital, Cincinnati, USA; 3 Information Services, Cincinnati Children’s Hospital, Cincinnati, USA; 4 Division of Dermatology, Cincinnati Children’s Hospital, Cincinnati, USA; 5 Department of Radiology, College of Medicine, University of Cincinnati, Cincinnati, USA

**Keywords:** Enterprise imaging, Medical photography, Photocapture, Photodocumentation

## Abstract

The widespread availability of smart devices has facilitated the use of medical photography, yet photodocumentation workflows are seldom implemented in healthcare organizations due to integration challenges with electronic health records (EHR) and standard clinical workflows. This manuscript details the implementation of a comprehensive photodocumentation workflow across all phases of care at a large healthcare organization, emphasizing efficiency and patient safety. From November 2018 to December 2023, healthcare workers at our institution uploaded nearly 32,000 photodocuments spanning 54 medical specialties. The photodocumentation process requires as few as 11 mouse clicks and keystrokes within the EHR and on smart devices. Automation played a crucial role in driving workflow efficiency and patient safety. For example, body part rules were used to automate the application of a sensitive label to photos of the face, chest, external genitalia, and buttocks. This automation was successful, with over 50% of the uploaded photodocuments being labeled as sensitive. Our implementation highlights the potential for standardizing photodocumentation workflows, thereby enhancing clinical documentation, improving patient care, and ensuring the secure handling of sensitive images.

## Introduction

The availability and convenience of smart devices have allowed a significant expansion in the use of clinical photography in medicine. Specialties such as dermatology or reconstructive surgery have historically utilized clinical photography. In this historical setting, healthcare providers or medical photographers used digital single-lens reflex (DSLR) cameras to capture a photograph. These photographs were often stored on a shared divisional network drive and inserted into a clinical note. As the quality and availability of smart device cameras has increased, other specialties have increasingly obtained photographs at the point of care [1–5]. In this modern setting, the use of clinical photography has expanded as the photograph is felt to be a more accurate and effective method of communicating and documenting information about a patient’s condition [6]. A photodocument is a digital photograph or set of photographs captured for medical purposes, integrated into the electronic health record (EHR) through a standardized workflow to document patient conditions, enhance clinical documentation, and support patient care. In combination with the clinical note, the images acquired are a major asset to patient care and further augment the utility of the electronic health record (EHR) [7, 8].

While there has been increased use of clinical photography, standardized workflows integrated within the EHR to acquire and store photographs are limited [9–11]. When these workflows exist, the images are typically inserted into a clinical note. While this workflow allows for images to be associated with a clinical note, it is not an ideal information technology solution and can lead to several downstream problems. For example, images inserted into a clinical note are associated with loss of image data, loss of image metadata, inability to recall images directly, and the inability to directly compare images obtained at different time points [12–15]. Ultimately, we believe that this type of workflow limits the power of the photograph to enhance patient health.

The purpose of this manuscript is to describe the development and implementation of a photodocumentation program at our institution. Key aspects considered during the development of the current program included safety and security, workflow efficiency, and routine application of key metadata elements for optimal viewing and image retrieval.

## Materials and Methods

### Setting and Infrastructure

This photodocumentation project occurred at a large academic children’s hospital. The main hospital system includes two hospitals and nine outpatient locations. The workflow was implemented across all phases of care including the ambulatory, emergency, inpatient, and perioperative settings.

The implementation project occurred under the guidance of the preexisting enterprise imaging program. The hospital uses an enterprise imaging archive (iConnect; Merative, Ann Arbor, MI) with an associated image viewer (iConnect Access; Merative, Ann Arbor, MI). The image viewer can be accessed directly or through the hospital’s EHR (Epic Systems, Verona, WI). Images are stored and can also be viewed through the picture archiving and communication system (Merge PACS; Merative, Ann Arbor, MI). An app (ImageMoverMD, Madison, WI) was used for all mobile photocapture and DSLR image upload. The application is integrated within the EHR and is smart device agnostic allowing use on iOS (Apple, Cupertino, CA) and Android (Alphabet, Inc, Menlo Park, CA) devices.

The institution has a large, established enterprise imaging program with the goal to build efficient workflows and enable viewers to “capture, index, manage, store, distribute, view, exchange and analyze all clinical imaging and multimedia content” [7]. The enterprise imaging governance system is comprised of executive, clinical, technical, and operations teams. The operational governance team is at the core and is involved with managing the day-to-day operations and provides practical guidance regarding implementation, design, and feasibility of projects and workflows. Other teams are consulted ad hoc to provide feedback or make decisions related to budget and strategy (executive governance), prioritization and clinical care (clinical governance), and technical solutions (technical governance).

### Principles Governing Photodocumentation Workflow Development

As the team developed the solution, they strived to build a workflow that utilized imaging informatics standards and minimized steps for image capture and viewing, enabled the application of standard metadata, and kept patients and their data safe while optimizing care. Each of these principles was challenged during the creation of the workflow.

At the outset, the team had to decide whether to store images in the acquired format or convert the image to the Digital Imaging and Communication in Medicine (DICOM®) standard. The DICOM standard was ultimately accepted because it enabled the application of standard patient, study, and image metadata. While this information could have also been applied using the Exchangeable Image File (EXIF) metadata fields associated with digital photography and utilizing the cross-enterprise document sharing (XDS) standard, there was an increased cost associated with this solution and a concern that other organizations would not be able to consume the images if they needed to be transferred.

**Fig. 1:**
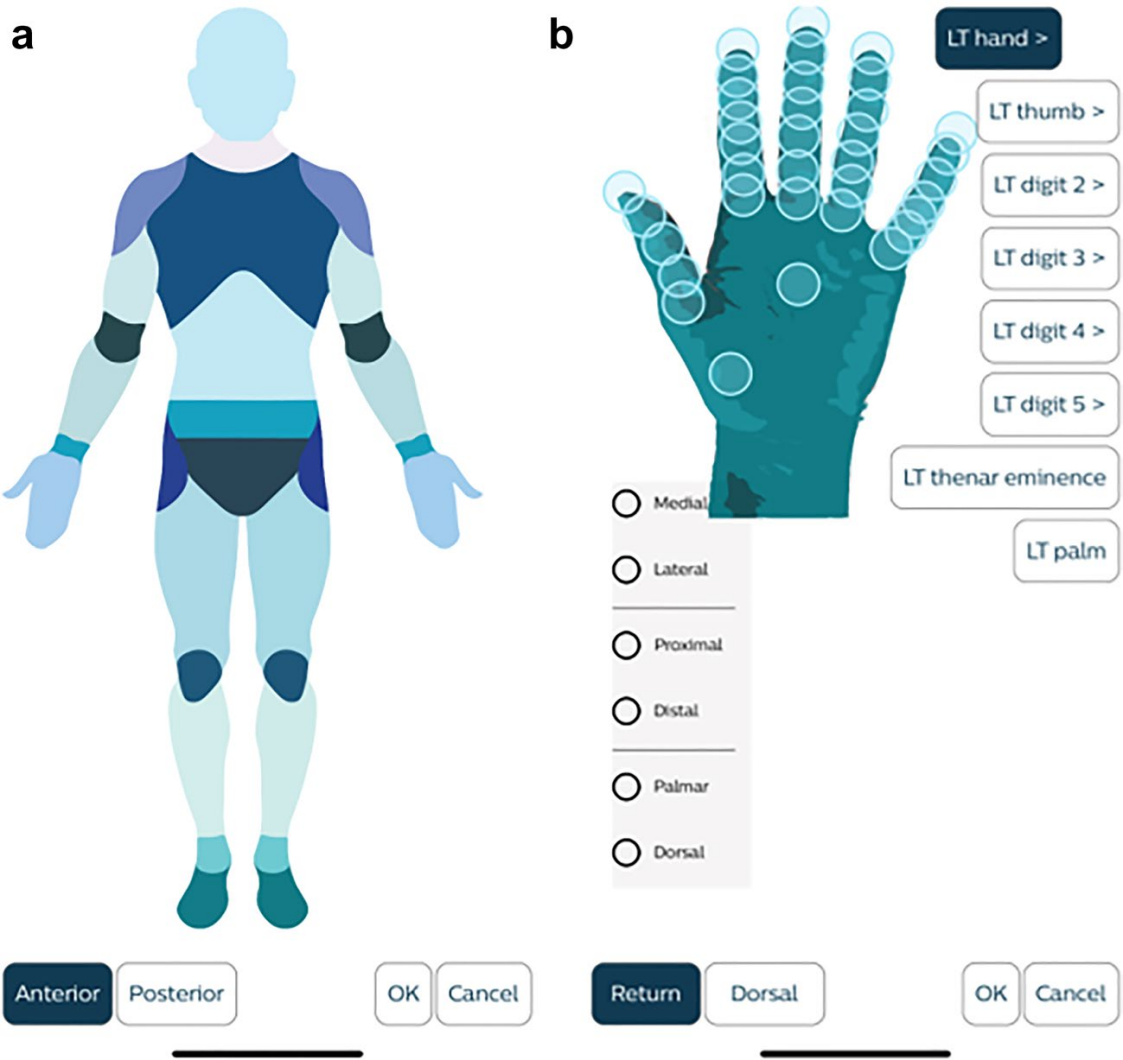
**a** Screen capture highlighting the body part map used within the photocapture app. **b** Once a body part is selected, a more detailed map is displayed for use, if desired. In this example, the detailed map of the hand is displayed. Screen capture from ImageMoverMD app (ImageMoverMD, Madison, WI)

For the image capture and image viewing workflows to be efficient, the team needed to standardize data elements. Key data elements that required standardization included modality code, procedure description, body part(s) imaged, medical specialty, and acquisition device. The clinical governance team helped to make decisions related to these elements. Table 1 summarizes the decisions made by the clinical governance team. Once body part terms were decided, the project team worked with the vendor to create a body part map that attempted to balance efficiency and the anatomic specificity needed for certain specialties. For example, a provider could select a large body part (right hand) with a single tap on the body map. However, if they required additional specificity (distal phalanx, right middle finger), the selection of the larger body part opened a more detailed body part map of the larger structure (Fig. 1).

**Table 1:** Summary of governance questions and decisions affecting the application of metadata for photodocuments

Governance question	Decision
Which DICOM modality code should be used as the Attribute Modality (DICOM tag: 0008, 0060) for photodocuments?	The External-camera Photography (XC) modality code was selected
Which DICOM SOP Class (DICOM tag: 0008, 0016) should be used for photodocuments?	The Secondary Capture Image Storage SOP Class was selected
What procedure description naming convention should be used for photodocuments?	The following naming convention was created: (SENSITIVE)-PHOTO-DEPARTMENT-BODY PART(S) where sensitive is a variable element, the department is determined by the department the user is logged into within the electronic medical record, and the body part(s) are those selected by the user
Should body part be a required selection?	Body part was determined to be a required selection for each image
Which body parts should be considered sensitive?	The face, chest, genitals, and buttocks should be considered sensitive
How is the medical specialty determined?	The medical specialty is determined by the department the user is logged into within the electronic medical record
When a user is logged into an inpatient unit within the electronic health record (rather than an ambulatory department), should each medical unit be identified separately or should all inpatient units be combined?	All inpatient units should be combined and labeled as “INPATIENT.”
Should we allow providers to use their personal devices?	Yes, the selected photocapture app provides users with a safe and secure way to capture photos. Other clinical applications are already allowed on personal devices
Should personal device names populate the DICOM Station Name Attribute (0008, 1010)?	No, the DICOM Station Name Attribute should be morphed to a common name indicating whether mobile or workstation upload was performed
Should we limit the use of the photocapture app to certain roles within the hospital?	No, all users should be able to capture patient images regardless of role
Should the photocapture app be installed on hospital-provided smart devices?	Yes, this would enable nursing staff, respiratory therapists, and other staff to obtain photos
Should photodocument image viewing be limited to certain roles?	No, limiting image viewing would lessen the impact of the photograph. Instead, the same privacy rules that apply to the electronic health record should apply to image viewing

The team believed that a simple and efficient workflow was key for adoption of photodocumentation. To this end, the team tried to automate as many steps as possible. For example, all patient metadata and most image procedure data were automatically associated with the photographs by scanning a quick response (QR) code generated by the image capture application embedded within the EHR at the initiation of the workflow. Other automated steps included the creation of links to the images within the EHR, the creation of the imaging study’s procedure description based on the body part(s) imaged, and the application of a sensitive image tag based on the body part(s) imaged. The creation of the link within the EHR occurs after the images arrive at the image archive. Once the imaging study is complete, a basic study content notification (BSCN) is sent from the archive to the EHR.

While most steps were automated, selection of the body part imaged was required as this data element was thought to be crucial for later image viewing and retrieval. Because many body parts could be imaged in a single imaging study, a body part “roll-up” schema was also created to simplify procedure descriptions. For example, if images of the left hand, forearm, and upper arm were all obtained as part of the same procedure, the study description would automatically be modified to the left upper extremity. In addition to creating a roll-up schema, the clinical governance team also decided that specialties could create photo sets. These specialty-specific studies were defined as a routine collection of photographs used to document a specific condition or indication. An example of this was the pectus photo set created by our chest wall deformity clinic. In this workflow, the clinic providers obtain the same five photographs of the chest (from the front, from the back, oblique left, oblique right, and with the patient bending forward).

Safety, security, and privacy were thought to be crucial for patient acceptance of a widespread photodocumentation workflow. First, there was a concern that healthcare providers would use the native camera app on their personal smart devices to capture images. While this workflow would allow for easier distribution of images (such as in secure text messages), the executive governance team determined that this workflow would be less secure and could represent a liability for the organization as many medical photographs contain protected health information. If images were captured by the device’s native camera app, they would be stored in the native photo app as well as any photo cloud associated with the device. To address this concern, the executive governance decided that images had to be captured within the hospital’s photocapture application (which deliberately does not store images on the device) and could not be uploaded from the device’s photo library. Next, the clinical governance team decided which body parts should be sensitive. These body parts would automatically apply a label in the procedure description and, when possible, would have additional protections applied [16]. The clinical governance team decided that all photographs of the face, chest, and pelvis (both from the front and from behind) should be labeled as sensitive.

During the development of the workflow, several governance decisions were required to optimize care. These decisions were all made by the clinical governance team and are summarized in Table 1. Several more consequential decisions are described below. First, the team decided if image capture and viewing should be limited to specific roles within the EHR or allowed to be used by all. The team decided on the latter, more open approach as it would enable all members of the healthcare team to photograph patients during care delivery. The team believed that restricting image capture and viewing would also limit its impact. Additionally, they believed that viewing of images should be governed by the same policies that govern viewing of other components of the medical record. The next decision to make was if the image capture application should be added to hospital-provided smart devices. These devices are primarily used by nursing staff at our organization. Again, in the name of maximizing the impact of medical photographs, the team decided to add the application to these devices.

### Assessment

The photodocumentation workflow was assessed for efficiency and utilization after implementation. To assess its efficiency, the number of clicks, taps, and swipes was determined from the decision to acquire the image until photo upload and application closed. A range of the number of clicks was determined for some steps to account for mobile device (e.g., number of steps to unlock a device), DSLR camera (e.g., image transfer to a computer), and patient variability (number and complexity of images acquired). During the determination of workflow steps, several assumptions were made. These assumptions included (1) the user had already used the application, (2) the user was already signed into the EHR, and (3) the patient’s record was already opened. These steps were assumed as (1) there are extra steps for first-time users (i.e., app download) and (2) there is variation in patient selection depending on the care setting and method of chart access.

To assess utilization, the number of photos uploaded per week was determined from the initial pilot phase go-live through December 31, 2023. The number of photodocuments obtained is presented in an annotated run chart. Standard process control chart rules were used to identify the median number of photodocuments obtained per week and determine how the median value changed with time. Additionally, the number of medical specialties that obtained a photo study and the volume of photography studies per specialty were collected to gauge the use across the system. Finally, the number of photodocuments marked as sensitive was also collected to determine the percentage of sensitive photodocuments obtained.

## Results

The photodocumentation workflow was first launched in November 2018. The initial go-live occurred in a pilot phase in a single ambulatory clinic. The pilot expanded to a single ambulatory division in February 2020, to all ambulatory divisions in June 2020, and then to all phases of care in February 2022. Key project milestones are included in Table 2.

**Table 2:** Number of photodocuments performed between key milestones and December 31, 2023

Milestone	Date	Number of photodocuments performed after key milestone
Pilot go-live	11/13/2018	31,897
Expanded pilot	2/17/2020	29,789
Added automated sensitive label	2/17/2020	29,789
Go-live for DSLR uploader tool	3/30/2020	29.622
Go-live for all Ambulatory divisions	6/9/2020	29,526
All images sent to radiology PACS	4/13/2021	26,163
Go-live for inpatient, emergency department, and perioperative settings	2/15/2022	21,522

**Fig. 2:**
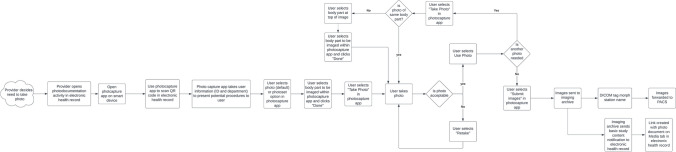
Photodocumentation workflow diagram

The workflow process map is provided in Fig. 2. The process starts with the decision to acquire a photograph. The healthcare worker opens the patient’s chart in the EHR and clicks the photocapture activity. They then open the photocapture application on their smart device and scan the QR code displayed within the EHR. At this point, the device is connected to the image capture server, associated with the logged-in user, and associated with the specific patient and the new imaging study.

After scanning the QR code, there are four fields to complete in the photocapture app. The first field is the “Study Description.” This field is defaulted to the description of “PHOTO.” This selection is used for most ad hoc photocapture but can also be changed to a predefined photo set from a drop-down list. The next field indicates if the image will be added as a new study or added to a preexisting study. This field is defaulted to be a “New Study.” Next, the image can be marked as sensitive by selecting a check box. This field is automatically checked if a sensitive body part is selected from the body part map. However, the healthcare worker can label any image as sensitive based on their discretion or patient preference. The last field is the body part to be imaged. This is the only required field that is not pre-populated or automated. The user must select this field to open the body part map where the region of the body to be photographed is indicated.

Once the fields are filled, the photograph is obtained. The image can be recaptured if needed and multiple images can be obtained in a session. When all the desired images are acquired, the images are sent to the image archive by selecting the “Send Images” button. Images are immediately viewable by accessing the image viewer directly or within 5 min after the last image arrives to the archive through the link that is created within the EHR. If the user accesses the images directly via the viewer, they have to manually search for the patient. If the user waits until the link is created, the link launches the viewer and takes the user directly to the photodocument.

The process for uploading images obtained via DSLR camera is similar. After image capture, the user selects the photocapture activity in the EHR. They click the media tab within the photocapture activity. From this tab, they click the “select files” button and then browse to find the location of the acquired images. Once the desired images are selected, the body part is applied using the body part map. The same sensitive body part rules apply. Like in the mobile app, the user can also elect to make a study sensitive by checking the sensitive images box. Finally, the user clicks the “submit files” button.

### Utilization and Efficiency Assessment

**Fig. 3:**
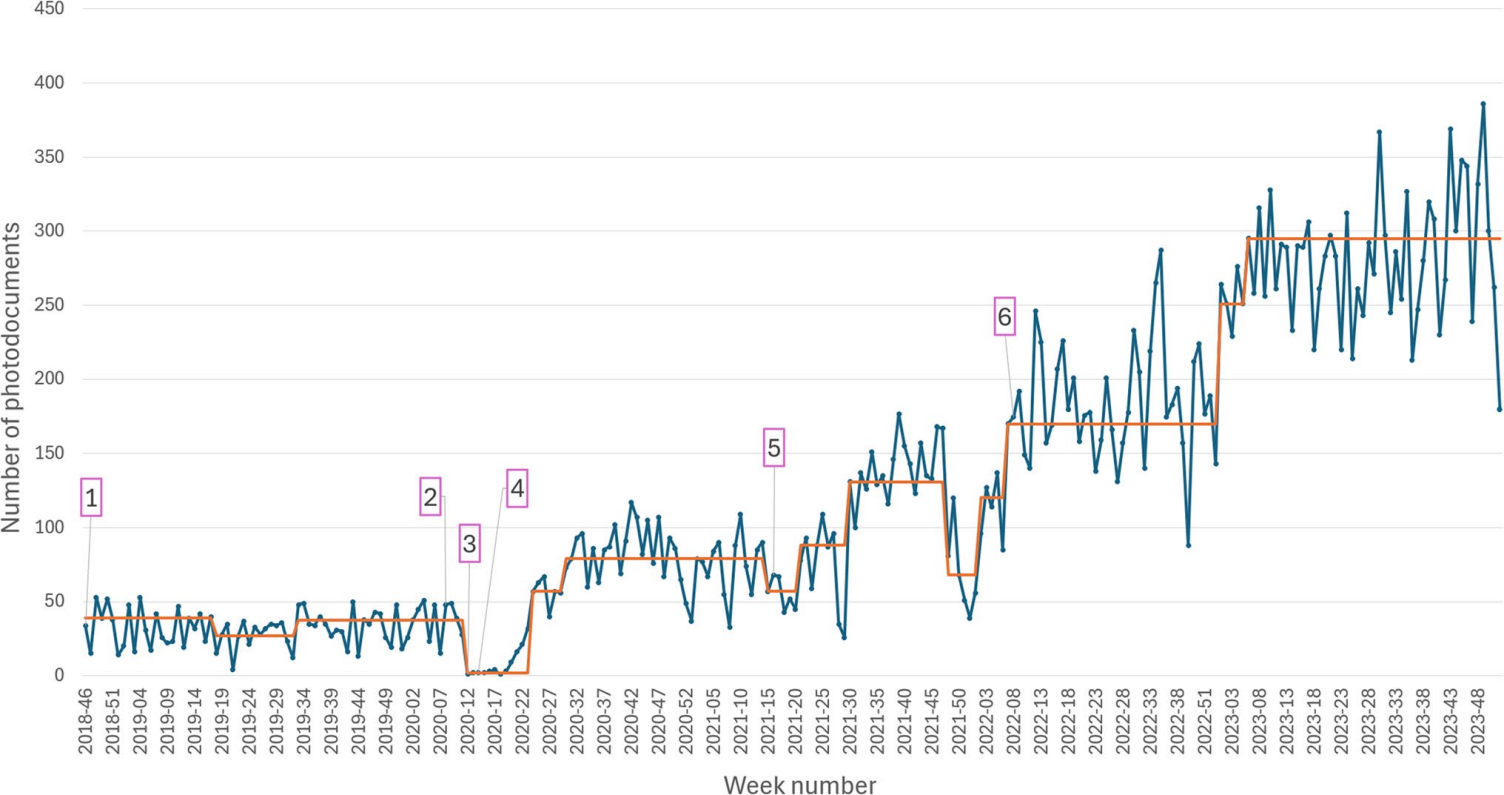
Annotated run chart showing the number of photodocuments uploaded each week from 11/13/2018 until 12/31/2023. The median number of uploads is displayed by the orange line. The shift in the orange line was determined using standard statistical process control rules. Specifically, the median was changed when 7 or more consecutive points were above or below the previously identified median value. Key dates are annotated: (1) pilot go-live; (2) expanded pilot and added feature to add sensitive labels based on body part; (3) start of COVID pandemic; (4) go-live for DSLR uploader tool; (5) photo sent to radiology PACS; (6) go-live for inpatient, emergency department, and perioperative settings

Between November 13, 2018, and December 31, 2023, there were 31,897 photo-based imaging studies uploaded (Fig. 3). The number of photo-based imaging studies performed from key timepoints is included in Table 2. We have been able to identify the method of image upload since April 13, 2021, when the photodocuments were sent to the PACS. Since then, approximately 70% (18,342/26,163) of all photodocument uploads used the mobile image capture workflow. Utilization increased throughout the observed timeframe. The number of photodocuments per week has continued to increase even after more widespread implementation. In 2022, there were on average 181 photodocuments per week for a total of 9440 photodocuments. In 2023, these numbers increased to 197 and 14,886, respectively. Since go-live, 54 medical specialties or divisions have uploaded at least one photodocument. However, 96% (30,511/31,897) of the photodocuments studies were submitted by 12 divisions. The volume of photodocuments per division for the top 12 highest volume divisions since the more widespread go-live on February 17, 2020, is shown in Table 3.

**Table 3:** Volume of photodocuments per division for the top 12 highest volume divisions since 2/17/2020 when automated sensitive labels were applied based on body part tags

Division	Number of photodocuments since 2/17/2020	Number of sensitive photodocuments since 2/17/2020 (% total division volume)
Pediatric Surgery	8168	7687 (94)
Dermatology	5768	1919 (33)
Plastic Surgery	4243	2106 (50)
Emergency Medicine	2109	833 (39)
Inpatient	1638	513 (31)
Hemangioma and Vascular Malformation Center	1308	266 (20)
Hematology and Oncology	1225	506 (41)
Genetics	1100	11 (1)
Colorectal	819	399 (49)
Gynecology	739	713 (96)
Craniofacial	655	206 (31)
Pediatrics	631	191 (30)

Of the captured photodocuments obtained since sensitive tags were applied, 53% were labeled as sensitive (15,884/29,789). Some specialties submitted a higher percentage of sensitive photodocuments than others. Of the top 12 highest volume divisions, the percentage of sensitive photodocuments submitted ranged from 96% (713/739) in Gynecology to 1% (11/1100) in Genetics. The number of total photodocuments and the number of sensitive photodocuments submitted for the top 12 specialties are included in Table 3. While we did not evaluate the reasons some specialties had a higher percentage of sensitive photos, we believe that it is related to body parts imaged rather than manual selection of a sensitive description. For example, in our Pediatric Surgery Division, most photographs are captured within our chest wall deformity or colorectal clinics. Because of the nature of these clinics, a high percentage of photographs of the external body are automatically labeled as sensitive.

The total number of clicks, taps, or swipes for mobile image capture was assessed starting from the patient EHR chart opened until the image capture application was exited (Table 4). The workflow requires at least 11 clicks, taps, or swipes. The number of steps could increase depending on the unlocking mechanism of the smart device, if fields needed to be changed from the default settings, what body part was selected, number of images to acquire, and if images needed to be retaken.

**Table 4:** The number of clicks, taps, or swipes needed to capture a photograph and send it to the imaging archive. The count was determined based on this being a non-first-time user of the photocapture application and having already logged into the patient’s chart within the electronic health record

Mobile photodocumentation steps	Number of clicks, taps, or swipes
Launch photocapture activity within EHR	1
Unlock smart device (variable based on device, locking mechanism, etc.)	≥ 1
Click photocapture application and scan QR code	≥ 1
Enter field *Study Description* or leave on default “Photo” (depending on if need to change default description)	0–2
Identify if photo is a sensitive image or not via checkbox	0–1
Select body part on map (taps depending on region such as hand and number of areas selected)	2–6
Tap *OK* to confirm the selection of body part	1
Tap *Done*	1
Tap *Take Photo*	1
Tap to capture photo	1
Tap *Use Photo*	1
Tap *Send Image* when complete	1
Total (minimum)	11

The total number of clicks and keystrokes for DSLR image capture and upload was also assessed (Table 5). This workflow requires at least 10 clicks and keystrokes. This number is likely undercounted. Additional clicks and/or keystrokes are often needed to find and select images within a computer’s folder structure. Additional clicks and keystrokes are needed if the images are transferred and/or removed from the camera’s storage media.

**Table 5:** The number of clicks and keystrokes needed to capture a photo using a DSLR camera and upload it to the imaging archive. The count was determined based on this being a non-first-time user of the photocapture application and having already logged into the patient’s chart within the electronic health record as the starting point. Additionally, steps required to transfer images from the camera to the computer were not counted as this could vary by camera and computer operating system

DSLR photodocumentation upload steps	Number of clicks and keystrokes
Obtain photo(s) on DSLR camera	1
Launch photocapture activity within EHR	1
Click *Media* tab	1
Enter field *Study Description* or leave on default “Photo” (depending on if need to change default description)	0–2
Identify if photo is a sensitive image or not via checkbox	0–1
Click *Select Files* button	1
Browse to folder containing photo(s) and select photos(s)	≥ 2
Select body part on map (taps depending on region such as hand and number of areas selected)	2–6
Click *Apply* to confirm the selection of body part	1
Click *Submit Files* when complete	1
Total (minimum)	10

## Discussion

The availability and quality of smart devices has made it easier for healthcare workers to obtain clinical photographs [4]. These clinical photographs can improve patient care as photographs are less subjective and provide richer contextual information than traditional text descriptions. In addition, photodocuments are more efficient to create and increase providers’ confidence in managing patients [6, 17]. Due to their accessibility and utility, photodocumentation is now used broadly by providers across medical specialties. The broad use was shown in a prior study which reviewed the percentage of photos contributed by a department or specialty. In that work, dermatology submitted the majority of photodocuments. However, photos were submitted by providers in emergency medicine, primary care, surgical specialties, and medical subspecialties [18]. At our organization, the majority of photodocuments were submitted by Pediatric Surgery, Dermatology, and Plastic Surgery. While 54 different divisions submitted at least one photodocument, 96% of all photodocuments were submitted by providers in one of 12 different divisions.

The benefits of photodocumentation have been established [2, 3, 6, 17]. However, there have been important challenges identified with implementing a photodocumentation workflow [4, 13]. A fundamental challenge involves a standard format of the image. Unlike traditional medical images, images from smart devices can vary and contain limited metadata. Without additional metadata, it would be difficult to efficiently recall and meaningfully compare prior images. It was decided that the native Joint Photographic Experts Group (JPEG) image files in our workflow would be DICOM-wrapped allowing additional metadata to be added, augmenting the image utilization for clinical purposes. The process of DICOM-wrapping the image was automatically performed by the photocapture application during transmission to the imaging archive.

When we implemented the image uploader tool, there was a discussion regarding the file format used for image storage. Our medical photographers preferred the lossless RAW format due to the fact that it has the least data manipulation [19]. Unfortunately, this file format could not be used as it is not standardized between companies and is not supported by the DICOM standard. File types supported by DICOM include JPEG, TIFF, and PNG. After discussion and testing, we recommended that the medical photographers upload lossless JPEG files [20].

Body part and specialty labels were used in the procedure description naming schema to guide workflow efficiency and image viewing effectiveness. Specifically, the body part and specialty information allow our end users to quickly understand the content and context of the photodocument. Though several other fields could be included in the naming schema, we did not think that they would add specificity to the study identification. This naming schema has laid the foundation for display protocols that automatically present relevant comparison images [21]. This benefit has already been realized by our radiologists. Anecdotally, they have appreciated the addition of photodocuments to their relevant comparison lists.

The requirement of photodocuments to have a body part label has allowed us to automatically identify sensitive images. Based on the body part, the majority of photodocuments stored at our organization are considered sensitive. We believe that this is the first work describing the percentage of sensitive photodocuments and one of the first to systematically apply a sensitive label to imaging studies. From the outset, we felt the need to protect our patients in every way possible. While a label included in the procedure description does not completely protect our patients, we agree with the recent HIMSS-SIIM Enterprise Imaging Community Whitepaper that stated that a label within the procedure description is a necessary first step until our technology standards can enable clinical systems to add permissions and preferences related to sensitive image viewing and sharing [16]. Until this is better addressed by the industry, we have worked with our viewer vendor to enable a pop-up warning when an imaging study with the word “sensitive” is opened.

Our photocapture application allowed us to maintain a simple and straightforward process of applying metadata without adding complexity to the process. While other workflows have been described, these workflows have either been custom developed or department-specific [18, 22–24]. We selected a vendor-based photocapture application so that we could have an organization-wide tool. We believe that this choice allowed us to create workflow efficiencies. For example, when users scanned the QR code within the EHR, the application automatically applied patient demographics and the user’s logged-in department to the image metadata. The automatic application of metadata helps to reduce errors that can arise from the manual entry of patient information.

The photodocumentation workflow developed at our institution is efficient with a minimum of 10–11 clicks/taps/keystrokes required from the decision to capture an image through the completion of the process. The simplicity of the process is also demonstrated by the quick adaptation of the workflow after the system-wide go-live in February 2022. We have seen continued increases in the system’s use over time. In 2022, a total of 9440 photodocuments were stored. This number increased to 14,886 photodocuments in 2023, a 55% increase year-over-year. While early increases in system usage were tied to the expansion of the application to different settings within the organization, further expansion did not occur in 2023. We attribute the increased utilization to word-of-mouth marketing as users continue teaching each other. We anticipate continued growth as new features are added such as automated photoset workflows and video capture.

There are several limitations of the workflow. First, the BCSN tool was retired by DICOM in 2006. While this status does not preclude its use, there are other options including the more modern Health Level Seven (HL7) Fast Health Interoperability Resources (FHIR) ImagingStudy Resource. Next, the images are captured using an encounter-based workflow. This decision was made to make the photocapture process as efficient as possible [25]. However, this decision has made it difficult to associate the appropriate clinical note with the images. This is especially true in the inpatient setting where many notes are created during the single inpatient encounter. Currently, we have placed the link to the photodocuments on the media tab within our EHR. This location is different than other imaging studies which reside on the imaging tab. We believe that this location makes it difficult to find the photodocuments. In the future, we hope to move the photodocuments to the imaging tab. It is possible that this change will be coupled with a conversion to an order-based workflow. Even if we make this change, we do not think that we will need to add workflow steps as we can create an ad hoc order based on the image metadata as described in the Integrating the Healthcare Enterprise’s (IHE) Encounters-based Imaging Workflow [26].

A third limitation of our workflow is that we do not allow users to upload images through the mobile EHR applications. While this mode of image upload is possible, it has several key deficiencies. Most notably, photographs obtained through the mobile EHR application are not able to be sent to the imaging archive. Because of this, photodocuments are not able to be compared over time and medical imaging metadata is not able to be applied. We believe that these deficiencies outweigh the benefit of users being able to insert a photograph into their notes. We urge the EHR vendors to create workflows that (1) allow the EHR mobile application to launch dedicated image capture applications within the patient context and (2) enable users to insert referential images into their notes via a tight integration with the image viewers. This type of workflow may be possible using DICOMweb-based image viewing hyperlinks as described in the IHE Interactive Media Report profile [27].

The fourth limitation of this workflow is that users are not able to use the photocapture app to send images via secure messaging. We purposefully do not allow users to upload images from their personal devices to our imaging archive. This governance decision was made to prevent users from storing patient information on their personal devices and in their personal cloud services. We believe that this decision is necessary to promote patient privacy. However, because the images are not stored on the device, they are not able to be sent via secure text message to other users. We acknowledge the importance of photography to convey a patient’s condition and recommend that vendors create solutions that allow users to communicate while simultaneously documenting and storing patient imaging data.

Finally, we note that we have not yet enabled mobile video capture. Video capture is associated with unique challenges and governance decisions. Enabling video capture is part of an ongoing project at our organization.

In conclusion, we have implemented an efficient photodocumentation workflow that leverages standards and allows users to capture, index, manage, store, distribute, and view photodocuments [7]. This manuscript highlights the potential impact of an enterprise imaging program to balance workflow efficiency, data fidelity, and patient privacy. Although aspects of the implementation are specific to our organization, many of the principles can be applied in other settings. Specifically, organizations should engage with their clinical governance to determine which photodocuments are sensitive. Additional efforts should be made to standardize procedure descriptions, body part labels, and medical specialty. If implemented efficiently, photodocumentation workflows have the potential to enhance patient care, medical documentation, and clinical collaboration across the healthcare spectrum.
